# Collectanea Medica: Consisting of Anecdotes, Facts, Extracts, Illustrations, &c.

**Published:** 1824-10

**Authors:** 


### COLLECTANEA MEDICA:

CONSISTING OF

### ANECDOTES, FACTS, EXTRACTS, ILLUSTRATIONS, &c.

*Relating to the History or the Art of Medicine, and the Collateral Sciences.*

Floriferis, ut apes, in saltibus omnia libant,

Omnia nos, itidem, depascimur aurea dicta.

---

ART. I.—*On the Use of the Tartar-Emetic Ointment in Epilepsy.* By JOHN CREIGHTON, jun. Esq. Member of the Royal College of Surgeons in Ireland.—(From the Transactions of the Association of the Fellows and Licentiates of the King and Queen's College of Physicians in Ireland.)

MANY of the children admitted on the establishment of the Foundling Hospital in Dublin, having been affected with epilepsy, the attention of the medical attendants was particularly directed to their relief; and, upon reading the account which Dr. Jenner gives of his success in curing several analogous complaints, by means of the tartar-emetic ointment, we were induced to make a trial of that remedy. For this purpose some of the worst cases were chosen: two of them rendered perfect idiots by the frequent recurrence of the disease, and others with such aberration of mind, and weakness of intellect, as to be but few degrees removed from the same degraded state. Though we have it not in our power to assert that a complete cure has been effected in any case, yet we have the satisfaction to state that the fits are now, in each patient, comparatively of rare occurrence, and are of so mild a character, as not to interfere with the health or strength of the individual. Indeed, these hitherto helpless objects are now capable of applying themselves to various useful employments, and are no longer a burthen to the Institution.

Each patient was allowed to remain a certain time in the Infirmary before the use of the ointment was commenced, both to enable us to ascertain the time of recurrence and duration of each fit, and also to submit them to an antiphlogistic regimen. This had no influence whatever on the disease itself, and was suggested to us merely from the plethoric habit of body which appeared in all of them, on their first coming into the Infirmary. The ointment made use of was in the same proportion as that used by Dr. Jenner in his cases, with some trifling variation.

The eruption produced by the friction of tartar-emetic ointment varied very much, as to its first appearance, in the different cases in which it was tried: in some it appeared in twenty-four hours, in others not for three or four days. The imperfectly-formed pustules, in some instances, were small, and at a distance from each other; in others, large and numerous; and, in two or three instances, they degenerated into painful, irritable blotches, which gave the patients some trouble for a day or

7

two, chiefly arising from the pressure or rubbing of the clothes against them. On this occurring, the ointment was immediately discontinued; but, with this exception, although we have used it extensively in various complaints, it has not been followed by any injurious effects, or been the occasion of tumors or sores difficult to heal.

The blotches sometimes produced by the ointment are rather painful for a day or two, when touched or rubbed. In a short time they are covered with several dry crusts, elevated above the surface of the cuticle, and lying one over the other. These have been called warty, cartilaginous elevations; and the successive layers of the crusts have been compared to the coats of an onion. They, however, very soon come off, leaving the parts underneath perfectly sound: in fact, if not interfered with, they will always heal of themselves. The simplest dressings are the best for them, while in the inflamed state; and the chief thing to be attended to, is to preserve them from friction. It is to be observed, that the eruption is not confined to the spot on which the ointment is rubbed, it most frequently appears in very remote parts; thus proving that its action is in some degree on the constitution.

Case I.—July the 11th, 1822. Catherine Hunt, admitted with epileptic fits, aged twenty-seven, was completely idiotic, and incapable of giving any information respecting her situation. Her nurse said that she had been regularly afflicted three or four times a day, in the most violent manner. She is very large and strong, but has never menstruated: she has not had epilepsy from infancy.

It appears that, during childhood, she was of a remarkably dull intellect; and, when arrived at a certain age, so viciously inclined, that it required constant watching and control to keep her within the bounds of propriety. She was apprenticed out of the Institution at the age of fourteen, but was in a short time sent back by her master, in consequence of her being attacked with epilepsy. From that time to the present, being a period of thirteen years, she has had the fits violently and constantly.

July 12.—Sixteen ounces of blood were ordered to be taken from her, and her bowels to be kept regular.

15th.—Frequent fits. The tartar-emetic ointment was now rubbed on her arms.

22d.—No recurrence of fits, but an appearance of fulness about the head. She was bled to the extent of twelve ounces, and the ointment was directed to be rubbed each night on different parts of her body.

26th.—No fits; but the ointment was discontinued, as the parts rubbed, particularly the back, between the shoulders, presented large inflamed blotches.

27th.—The back not so sore; the blotches covered with dry crusts. Simple dressings ordered.

29th.—The back nearly healed. Has been free from fits.

August 1st.—The ointment was now directed to be rubbed on the thighs. No fit.

13th.—Ointment continued. No fit.

14th.—Appears dull and heavy. Twelve ounces of blood directed to be taken.

21st.—Ointment discontinued, in consequence of great soreness. One slight fit.

September 12th.—Up to this period no fit. On the morning of the 13th, she had a fit of short duration. The ointment to be again used.

20th.—The ointment discontinued.

21st.—One short fit, the first for the last eight days.

23d, 25th, 28th, 30th.—She had one slight fit each day. On the 5th October, she had two fits, but by no means severe. The ointment resumed.

15th.—No fit since the 5th. But she has again become heavy and stupid. Ordered to be bled to the extent of twelve ounces.

On the 18th, one short fit. On the 24th, she was again bled.

28th.—Two severe fits. It is now twenty-eight days since she had any repeated attack. We are inclined to think that they are influenced by some effort to menstruate. She is placed for some hours daily in a hip-bath. Arms again rubbed.

30th.—One fit while in bed this morning.

She has now become much more rational. She is lively and animated, and anxious to be employed. At her own desire, she works daily in the laundry, and cheerfully discharges any duty allotted to her. She is clean in her person, and her appearance and habits have undergone a complete reformation.

Up to the 10th of March, 1823, when she returned to the country, she has had occasional recurrences of the epileptic attacks, once in three weeks or once a-month, but of such short duration, as not to interfere with or prevent her following her daily occupations. In July last, we heard from the person with whom she is now living, that she had the fits at times, but that she did not mind them, and had become an industrious and hard-working servant.

Case II.—John West, aged seventeen, has had epileptic fits from infancy, occurring with great violence six or eight times in a fortnight. He appears nearly idiotic; his hearing is impaired, and his intellect dull; his limbs are weak, and he walks with difficulty. As it would be unnecessary to detail the treatment in each of these cases, it being nearly the same in all, we shall only give the general appearance of the patients on their admission, and their present state of health.

This patient used the ointment, with occasional intermissions, till November, 1822. He has perfectly recovered the use of his limbs. He attended daily in the school, and is now so far educated, that he reads and spells well. He had a fit once a-month, or once in six weeks. He returned to the country in March, 1823; and is now, we understand, although subject to occasional slight fits, a strong and healthy boy.

Case III.—Michael Franklin, aged nineteen, has been subject to epileptic attacks as long as he can remember. They come on three or four times in a month, leaving him in a state of stupor for several days afterwards. His intellect is not impaired, but he seems incapable of making any exertion. He used the ointment during four months, with occasional intermissions. He is now so far relieved as to be employed in the garden of the establishment. He has a fit once a-month; but it lasts only a short time, and he immediately recovers from its effects.

CASE IV.—William Ord, aged nineteen, says that he has epileptic attacks once or twice in a fortnight; that they continue a long time; and that, before they commence, he generally feels a pain at each side of his head, immediately under the parietal bones. He appears healthy and strong in other respects, and is in full possession of his intellectual faculties. On the day of his admission he had a severe fit, which lasted upwards of twenty minutes, and was succeeded by the loss of strength and memory, which continued for some time afterwards. He commenced using the ointment, and continued under treatment for two months. He was apprenticed as a servant in February, 1823, and had not a fit for two months before he left the Institution.

CASE V.—Mary Lynch, aged fourteen, has had epilepsy for several years. The fits come on generally once a fortnight, and are repeated, with little intermission, for the space of one or two days, being always preceded by a pain in the head a day or two before the accession, and followed by great weakness. On her admission, she was dull and stupid, the fits being very violent and frequent. She has now become lively and intelligent: the fits do not occur oftener than once in two months; and on one occasion she has had not a fit for three months. She is at present employed as a servant in the establishment.

CASE VI.—Henry Woodgate, aged twelve, was almost in a lethargic state on his admission. He used to sit for hours in the same posture, and was roused from it with difficulty. He had epileptic fits three or four times in a-day; the intellect was obscured; and he appeared to be fast approaching to a state of idiotcy. Though the disease resisted for a long time this plan of treatment, yet its progress was at length so far arrested, that he became capable of instruction: he attends in the school, and his teachers say he is by no means dull or stupid. The fits come on once or twice a fortnight, and they never recur on the same day.

Besides the above, there are several other cases at present under treatment, and which are progressively improving.

The tartar-emetic ointment has been used at the Foundling Hospital in every case of hooping-cough, during the last year, with evident good effect: the paroxysms were diminished not only in violence and frequency, but the progress of the disease so much shortened, that in no instance did it continue more than three weeks. The application of the ointment in these cases was along the course of the spine.

---

ART. II.—*On the Pathology of Epilepsy.* By ROBERT REID, M.D. Licentiate of the King and Queen's College of Physicians in Ireland. —(From the Transactions, &c.)

MEDICAL science has made such rapid advances within the last few years, that an immense number of important facts have been developed relative to the nature of diseases. Though the human mind may be incapable of appreciating the modes of action of ultimate causes, yet the laws which are obeyed in the animal frame are fixed and immutable.

Many of the errors, both in the theories of diseases and in the modes of treating them, may be attributed to physicians mistaking effects for causes. In no instance is this more conspicuous than in the history of the disease called Epilepsy. Although the enumeration of writers who have treated on this subject would occupy a volume, yet the last elaborate author (Dr. Cooke) is compelled to acknowledge, that much is still to be known before the true nature of the disease can be understood.

About eight years ago, when attending the hospitals of the House of Industry, with the late Dr. Edward Percival, our attention was particularly directed to ascertain the efficacy of spirit of turpentine as a remedy, at that time recommended for the cure of epileptic diseases. While studying the phenomena incidental to such a formidable disorder, I felt convinced that an extensive field for investigation lay yet untried, notwithstanding all that had been written on the subject.

Early in the year 1817, I laid before the public a treatise on Tetanus and Hydrophobia, in which it was stated, that the moving powers of the human frame are under the influence of the nervous mass situated in the spinal column, and that they hold to that mass as to a reciprocal centre. This idea is now very generally adopted. In considering the phenomena of epilepsy, a derangement of the moving powers forms the first and most characteristic feature of the disease: it, therefore, should be ranked among those diseases to which what I have denominated the *spinal system* is liable.

Pure epilepsy is very seldom fatal. In thirty-four cases which came under my notice, two only died of the disease, who, on examination after death, exhibited no morbid appearance sufficient to account for death, until the spinal column was opened along the cervical vertebræ, when the membranes enveloping the medullary mass appeared covered with a minutely injected vascular tissue.

The morbid states of other parts, particularly in the head, which have been the immediate cause of death in cases previously subject to epilepsy, can only be considered the consequences; for similar states have been met where no such disease attended. It is important to be aware, when investigating the diseases incidental to the spinal system, that they frequently run into each other. Thus a person, while under treatment for epilepsy, may suffer tetanic spasm, then chorea, then catalepsy, &c. A case of this kind occurred to me not long since, of which the following is an abstract:—

June the 8th, 1818.—Mr. ——, ætatis eighteen, subject to epilepsy; has no apparent malformation of the head. Has been several years affected with the disease, the commencement of which he attributes to fright. Remarks that his memory is of late very much impaired. The fits are very frequent, sometimes twice a-week, sometimes three times in the same day. Is generally attacked early in the morning, without any previous notice, or a sensation of cold in any part of the body. The pulse is very contracted, irritable, and quick. I determined, if possible, to see him during the paroxysm; and in five days after, (13th of June,) on visiting him about eight o'clock in the morning, when he was dressing, I found that there was not the slightest pulsation at either wrist, which previous observation in other cases enabled me to conclude was the indication of an immediate attack. In two minutes afterwards he fell in the fit. The upper extremities were particularly affected, with general tendency to emprosthotonos. When the spasmodic actions had almost subsided, the pulse became enlarged, soft, and regular. The next fit was on the 19th of June: he was ordered a blister between the shoulders, over the spine, with directions to keep it open for some time; but he allowed it to heal, and had another fit on the 26th. He, however, appeared to improve in every respect.

A fit occurred on the 1st of July, when he was ordered a purgative, with spirit of turpentine, to be repeated every morning. This, at first, seemed to induce giddiness and sickness at stomach. It caused copious evacuation from his bowels, and a frequent desire to pass urine, attended with some smarting and irritation at the extremity of the urethra.

July 18th.—Since 26th June he has had no fit. The irritation of the urethra still continues severe. A few drops of blood are discharged after passing urine. The quantity of turpentine has been gradually lessened to five drops every morning.

August 1st.—The effects of the turpentine have, for some time, entirely ceased, and he had a fit yesterday. He could not be persuaded to continue the turpentine.

August 30th.—Has had several fits since the 23d, and his mental powers are very much impaired. This evening, he is delirious at intervals, and complains of severe pain following the course of the nerves of the lower extremities, with frequent sensation of cold along the spinal canal, sometimes ascending towards the head. He then feels violent pain in the occiput, extending towards the central regions of the head. He soon after speaks delirious. During one interval, he mentioned that he distinctly saw a crab upon his pillow; but that he knew it was only imaginary, yet could not prevent such an impression on his vision.

August 31st.—Has frequent tetanic spasms of various muscles, but no difficulty of swallowing. The cerebral affections have been relieved by leeches and cold affusion to the head, and active purgatives. During the operation of the purgative medicine, he had strong tetanic spasms of the parts which were usually affected in his epileptic paroxysms. Has no pain of head; perspiration copious; tongue white in the centre; pulse quick, but soft and regular. The tendons of his wrists and arms remarkably tense. Has now some inclination to sleep.

September 1st.—Remained easy during the night; has had slight cramps in the lower extremities, and is often affected with convulsive laugh, which he says, he cannot avoid, though he knows no cause of it. Pulse quick; head hot; tongue furred. Leeches were applied to his temples, and the cold affusion to his head; soon after which, the above symptoms ceased, and he inclined to sleep. His father reported to me, that last night, while at the night-chair, he became completely cataleptic, but retained the power of vision and hearing. His father desired him to endeavour to move his fingers, at the same time putting his own in a similar motion. After some apparent internal exertion, he succeeded. His father then moved his wrists, and persuaded him to try that also; after similar exertion he performed that movement; and so one part after another, until he recovered.

September 3d.—No return of cramp; skin soft and moist; rigidity of the tendons gone; hearing acute; vision wonderfully improved, so that a glass, which he has been for some time obliged to use, is not required, and does not now match his sight. His memory and other intellectual powers are natural. Feels his head get giddy after speaking much, and strong light is unpleasant.

September 4th.—Feels well. Has occasionally, for the last two days, a disagreeable sensation of cold in his feet, with inclination to shivering, which goes off by perspiration.

He continued convalescent, without any return of fits, till the middle of September, when he went for change of air into the country. I think it more than probable, however, that, after some time, he may have had a return of the epileptic fits.

I have made choice of the above case, as being the most remarkable for the number and variety of the changes from one spinal disease to another. It is at present impossible to determine the true seat of epilepsy, by the morbid appearances detected in epileptic patients after death: it is, therefore, necessary to combine with them the train of operations which occur during a paroxysm of the disease. By such investigation, the probable sources of the morbid phenomena may be discovered. In all the cases I have had an opportunity of observing, the rapidity of the changes in the morbid actions during the paroxysm, often differed very much; but the regular succession in which they followed each other was invariable.

As this disease must have been the same in the earliest periods of its occurrence that it is found to be at the present day, I shall refer to the actual operations, as they occur before our eyes, rather than to any written authority on the subject. This I am the more inclined to do, as the unfortunate frequency of the disease may enable every practitioner to judge for himself.

By considering the phenomena according to the regular train in which they are developed, it would appear that each succeeding occurrence is the natural consequence of that immediately preceding. It will be found, on careful attention, that the first symptom of an attack is the suspension of the action of the heart, and consequently an intermission of the pulse, which may continue from a few seconds to about three minutes, which was the longest period of intermission I have as yet known.

The *aura epileptica* can only be considered a premonitory symptom; for, in many cases, it never occurs. If all the other powers which contribute to circulate the fluids in the animal frame became quiescent at the time the heart ceased its activity, the well-known phenomena of fainting would be the consequence. But, during the epileptic quiescence of the heart, it appears that an accumulation of blood is taking place in another direction; for immediately, often instantaneously, a tetanic rigidity of the entire frame succeeds, during which the air is sometimes so suddenly expelled from the lungs, that the patient utters a piercing shriek. I have seen patients hop five or six times on both feet, with their bodies perfectly rigid, before they fell. It has been attempted to prove in the Treatise before referred to,* that the tetanic rigidity of all the muscles of the frame was consequent upon accumulation of blood, or other irritation in the spinal nervous mass. That this is the case in the present instance, is confirmed by the examination of those cases which have proved fatal after only a few paroxysms, and before the injurious effects of the disease were conspicuous in other parts. When this accumulation amounts to compression of the nervous structure, the patient falls, and all the parts are for a moment relaxed. The animal economy now imperiously requires the function of respiration, which had been suspended during the phenomena before mentioned; but this suspension of respiration was gradually causing an accumulation of blood in the head, so as to bring the brain into an apoplectic state, by which means the control of the brain over muscular action is interrupted. Hence arise the irregular actions of the complicated organs of respiration, forming those convulsive struggles which are so peculiarly characteristic of this disease.

It is at this period that the unfortunate patient may have the paroxysm almost instantly terminated by mechanical means. Though it may be more proper to defer stating any mode of relief, until speaking of the treatment of the disease in general, yet the means alluded to tend to elucidate so much the nature of the convulsions, that they could not with advantage be omitted at present. Careful observation in a number of cases has convinced me, that there are two modes by which the convulsions may be suppressed, until the brain recovers influence sufficient to direct the muscular activity. During the inordinate struggle to perform respiration, the practitioner may abstract some of the force applied to the respiratory organs, by attracting the exertion in another direction. Thus, while the hands and arms are violently contracted, if the attendants forcibly extend them, and open the fingers, so much exertion is involuntarily made by the patient to oppose this, that the violent operation of the respiratory muscles subsides, the organs fall into their natural train of action, the patient draws a heavy sigh, and the paroxysm is at an end. Any unusual irritation may have this effect; among others, that which is stated to have been tried by Dr. Pickels, in his important paper "on the Discharge of living Insects from the Stomach." The other mode of putting a stop to the paroxysm, as far as I have experienced, is much more powerful, as it operates by suppressing the powers which excite the muscles into action. The peritoneum appears to be connected by the strictest sympathy with the nervous apparatus of the spine; for, when tetanus takes place, the tension of the peritoneum is one of the first remarkable symptoms; and it is well known what extreme debility attends contusion, inflammation, or other injury of this membrane.

When making experiments some time ago, for the purpose of ascertaining what part of the animal frame was particularly acted on by nux vomica, when taken in excess, I found that the animals (rabbits and dogs), in a short time after receiving the poison into their stomachs,

---

* Vide *Treatise on Tetanus and Hydrophobia.*

became tetanic. During the spasm, I observed that the peritoneum seemed closely to invest and compress the contents of the abdomen. Upon pressing forcibly a part of this membrane between my fingers, for the purpose of detaching one portion of it, so as to relieve the supposed compression of the bowels, I was rather surprised to find the spasms totally relax, and the animal begin to breathe, as if recovering from much fatigue. The moment the peritoneum was let loose, the spasms returned with violence; and this could be repeated at pleasure.*

When reflecting upon this curious phenomenon, it appeared to me that, were it possible to afford the necessary compression of the peritoneum in the human subject, while labouring under a paroxysm of epilepsy, that the fit may be as instantaneously cut short. Opportunities were not long wanting for putting this operation into practice, and it was attended with the utmost success. The manner in which this may be accomplished, is by pressing the closed hand of an assistant forcibly on the soft part of the abdomen, towards the spine, while the patient is firmly supported on the back, with the head and shoulders raised. While this operation is performing, the practitioner will often perceive a very peculiar flapping of the diaphragm, without apparently contributing to the purposes of respiration. This I have most usually met in puerperal convulsions. When it is considered that the consequences of epilepsy are still more dreadful than the disease itself, and that the most severe of these, such as idiotism and insanity, are caused by the effects of the disease upon the cerebral structure, it cannot be doubted how important to the community at large any mode would be which could thus cut short the paroxysms, and obviate those injurious effects upon the brain.

I have endeavoured to show that the symptoms which occur during a paroxysm of epilepsy follow an invariable course, each depending on the other as cause and effect. It has also been perhaps sufficiently proved, that the immediate cause of a paroxysm of the disease should be attributed to a morbid accumulation of blood upon the nervous mass of the spine.

Having advanced so far, perhaps at some future period the observation of Celsus may be found applicable to the circumstances of this disease, when he says, "Et causæ quoque æstimatio sæpe morbum solvit."

---

* It may be worthy of observation, that, after repeating the above experiment of alternately compressing and letting go the peritoneum several times, the animal at length ceased to be affected by spasm; although, upon killing it, the poison was found still remaining in the stomach and small intestines.

